# The relationship between the perceived personalities of dating partners and dating violence victimization: A three-month longitudinal cross-lagged panel study

**DOI:** 10.1371/journal.pone.0344975

**Published:** 2026-03-17

**Authors:** Kohei Koiwa, Daisuke Kobayashi, Fumi Seki, Ayaka Uchiyama, Nami Ishigaki

**Affiliations:** 1 Graduate School of Education, Hokkaido University of Education, Sapporo, Hokkaido, Japan; 2 Graduate School of Clinical Psychology, Niigata Seiryo University, Niigata, Japan; 3 Tanino Gozan Hospital, Wakeikai Medical Corporation, Toyama, Japan; 4 Faculty of Education, Graduate School of Tohoku University, Miyagi, Japan; 5 SoftBank Corp., Tokyo, Japan; Caleb University, NIGERIA

## Abstract

Dating violence is a significant social issue with serious psychological consequences. Victims’ perceptions of perpetrators’ traits may both influence and be influenced by abuse. However, most existing studies are cross-sectional, providing limited insight into the temporal and reciprocal relationships between these variables. Therefore, this study aimed to examine the directional and reciprocal associations between victims’ perceptions of perpetrators’ personalities and their experiences of dating violence. A three-month longitudinal survey was conducted with 206 young adults (aged 18–29 years) who were currently in romantic relationships. Three personality characteristics previously linked to dating violence perpetration were assessed: the basic Big Five personality traits, aggression, and attachment style. We used a cross-lagged panel model (i.e., a statistical approach that estimates directional and reciprocal influences over time) and found three key results. First, among the Big Five personality traits, perceived “agreeableness” exhibited a cross-sectional relationship with dating violence victimization. Second, perceived aggression had a bidirectional effect: viewing partners as more aggressive predicted later victimization, and dating violence victimization predicted higher subsequent perceptions of partner aggression. Third, perceptions of partners’ “fear of abandonment” were shaped through experiences of dating violence. Among these findings, the bidirectional link between perceived aggression and victimization was the most robust and theoretically novel. These results suggest that victims’ perceptions of their partners’ anger-related traits predict and reflect abuse, underscoring the need for interventions that target mutual perception dynamics in violent relationships.

## Introduction

Violence in premarital relationships, also known as dating violence, is a major social issue. The concept of dating violence evolved in the late 1980s, expanding the concept of violence between married partners to include unmarried couples [1]. In Japan, violence between spouses or intimate partners had long been viewed as a private matter rather than a social concern [2]. However, the 2001 enactment of the “Act on the Prevention of Spousal Violence and the Protection of Victims,” and Yamaguchi’s [3] introduction of the concept of “date DV”—referring to behaviors resembling domestic violence but occurring within dating relationships, a term uniquely used in Japan—drew attention to violence not only within marriage but also in non-marital dating relationships.

### Prevalence and consequences of dating violence

Several studies have investigated the prevalence of dating violence perpetration and victimization. A review of international research revealed that among couples currently dating, 20–37% of high school students and 20–30% of college students face the risk of perpetration or victimization [4]. A government survey in Japan reported that 12.0% of men and 22.7% of women have experienced victimization [5]. Dating violence has many consequences for victims. Mental health problems, including depression, can worsen [6], which can increase the risk of suicide [7]. Beyond mental health concerns, multiple health risks and dependency on alcohol or drugs have been noted [8]. A social survey in Japan reported that the most frequently reported subjective change among victims was “loss of self-confidence” (27.0%), followed by “inability to sleep at night” (15.0%) and “physical and/or psychological distress” (15.0%) [5]. Collectively, these findings indicate that dating violence can have far-reaching and severe consequences.

In response, psychological research has explored the factors that contribute to dating violence. According to Akazawa [9], traditional research on dating violence has considered three types of factors: (a) individual factors, such as the perpetrator’s or victim’s personality; (b) relational factors, such as power dynamics between couples experiencing dating violence; and (c) social background factors, such as the social attributes and family environment of perpetrators or victims. Among these, research has primarily focused on perpetrators’ personality factors [10].

### Research on perpetrators’ personalities

The Big Five personality traits constitute a foundational personality framework frequently discussed in relation to dating violence and aggressive behavior. This theory conceptualizes personality across five dimensions—extraversion, agreeableness, conscientiousness, neuroticism, and openness to experience—and is widely recognized as a principal model of personality structure [11]. As a central theory, it has been extensively examined in connection with various aggressive behaviors, such as bullying [12], prejudice and discrimination [13], and domestic violence [14]. Theoretically, aggressive behavior is negatively correlated with agreeableness and conscientiousness, and positively correlated with neuroticism [15]. Low agreeableness is a strong predictor of aggression because distrust of others can lead to aggressive behaviors in conflict situations [16,17]. A recent systematic review [18] noted that most studies examining the associations between the Big Five personality traits and intimate partner violence perpetration have relied on cross-sectional designs, making it difficult to draw causal or reciprocal inferences. This suggests that further longitudinal research is needed to clarify the dynamics involved.

Research has examined the association between the Big Five personality traits and dating violence. For example, Hines and Saudino [19] found that high extraversion, high neuroticism, low agreeableness, and low conscientiousness in perpetrators were each associated with dating violence. Similarly, Carton and Egan [20] reported that low agreeableness among perpetrators is linked to dating violence. Thus, despite the Big Five being a fundamental theory of personality, it is relevant to the occurrence of dating violence.

Another group of psychological traits widely discussed in relation to dating violence involves anger-related tendencies. Müller’s Anger Coping Questionnaire (MAQ) conceptualizes four distinct modes of anger-related coping: aggression (external expression of anger), controlled affect (regulated suppression of anger), guilt (emotional response directed inward), and social inhibition (tendency to withhold anger in social contexts) [21,22]. Among these, aggression, defined as the outward and sometimes impulsive expression of anger, has received the most attention as a predictor of dating violence. Numerous international and Japanese studies have consistently linked this form of aggression to a higher risk of perpetration [23–26]. A systematic review of longitudinal studies on dating and intimate partner violence [27] reported that behavioral and emotional traits, such as aggression and poor emotion regulation, consistently predict later perpetration of violence in romantic relationships.

Another factor drawing attention in recent dating violence studies is perpetrators’ attachment style, which is widely used to explain behavior in intimate interpersonal relationships during late adolescence and adulthood [28]. It refers to individuals’ beliefs and expectations regarding the self and others, formed through developmental processes [29]. It encompasses two key dimensions: “fear of abandonment,” which refers to anxiety about potentially being abandoned by others, and “avoidance of intimacy,” which is defined as a tendency to resist close emotional involvement [30]. Theoretically, individuals with high fear of abandonment seek to control their partners more strongly, potentially leading to the perpetration of dating violence [31]. Lee, Reese-Weber, and Kahn [32] found that perpetrators’ unstable attachment characteristics are associated with perpetration. Similarly, in Japan, studies have linked perpetrators’ fear of abandonment to dating violence [26,33]. Longitudinal research has also been conducted to investigate this association. Miga, Hare, Allen, and Manning [34] reported that fear of abandonment predicted both victimization and perpetration of dating violence four years later, and a Japanese longitudinal study similarly showed that perpetrators’ fear of abandonment predicted their perpetration of dating violence six months later [35].

### Victims’ perceptions of perpetrators’ personalities

Despite the accumulation of knowledge regarding perpetrators’ personalities in the context of clarifying the mechanisms of dating violence, recent attention has turned toward how victims perceive perpetrators’ personalities. In social psychology research on personality perceptions, considerable work has examined how individuals infer others’ personalities. Studies have investigated the formation of personality inferences [36,37] and the factors contributing to individual differences in these inferences [38,39], often using experimental methods in hypothetical first-encounter scenarios.

More recently, researchers have sought to apply the concept of personality perceptions to clinical problems. For example, Kamphuis, Emmelkamp, and de Vries [40] explored the personality traits of stalking perpetrators from the victims’ perspective, whereas Spitzberg and Veksler [41] examined stalking perpetrators’ personality disorder characteristics from the victims’ perspective. Their findings revealed that victims often perceived borderline personality traits in their perpetrators. In Japan, Kobayashi and Wakashima [42] investigated the link between stalking victims’ perceptions of perpetrators’ personalities and victims’ emotional states, revealing that victims experienced strong distress when they perceived perpetrators to be highly neurotic.

Studies on dating violence have also investigated perpetrators’ personalities as perceived by victims. Koiwa, Uchiyama, Seki, Ishigaki, and Wakashima [43] examined how victims perceived perpetrators’ personality traits, finding that perpetrators were often viewed as aggressive, low in agreeableness and conscientiousness, and high in neuroticism.

### Shortcomings of previous research and the purpose of this study

Despite the importance of victims’ perceptions of perpetrators’ personalities, few studies have addressed this topic, and the directional and reciprocal influences remain unclear. Considering that some aspects of perpetrators’ personalities can predict future violence [35], it is reasonable to speculate that victims’ perceptions of these traits may predict future victimization [43]. Research in this area typically posits a unidirectional path from personality perceptions at one point in time to future dating violence victimization. However, if one considers that personality perceptions are formed through communication [44], the opposite causal relationship is also possible, in which experiencing dating violence causes victims to form particular perceptions of perpetrators’ personalities. These two possibilities suggest a bidirectional relationship: personality perceptions may predict future dating violence, while dating violence victimization may, in turn, shape personality perceptions. Despite this possibility, research on personality perceptions in dating violence remains limited to cross-sectional studies [43], with no investigations focusing on temporal influences or causality.

Therefore, this study aimed to examine directional and reciprocal relationships between dating violence victimization and perceptions of perpetrators’ personalities. To investigate these dynamics, a short-term longitudinal study was conducted using a cross-lagged panel model [45], allowing for the examination of reciprocal directional paths by analyzing multiple variables at several time points. This study focused on three personality characteristics that have been linked to the perpetration of dating violence: the basic Big Five personality traits, aggression, and attachment style, which has been the subject of recent research. A three-month longitudinal investigation was conducted to assess the relationship between these personality perceptions and experiences of dating violence. By testing a model that included not only paths from victimization to personality perceptions but also from personality perceptions to victimization, this study explored the directionality of these associations.

## Materials and methods

### Participants and procedure

Participants aged 18–29 years were recruited through a crowdsourcing service operated by CrowdWorks, Inc. At the first wave (T1), 1,050 individuals who reported having a current romantic partner participated in the survey (369 men, 680 women, 1 unspecified; mean age [M_age] = 24.34, standard deviation [SD] = 3.22). The three-month interval between waves was selected based on previous evidence that dating relationships undergo rapid relational and emotional changes during the early stages. Shulman, Tuval-Mashiach, Levran, and Anbar [46] found that most late-adolescent couples who exhibited conflictive interaction patterns dissolved their relationships within three months, suggesting that this period represents a critical window for observing the emergence and escalation of relational conflict and violence. Moreover, Hesse, Shorey, Brem, Stuart, and Cornelius [47] conducted a short-term longitudinal study over approximately three months and demonstrated meaningful changes in dating violence perpetration among female college students, indicating that this time frame is sufficient to capture the dynamic variations in relationship aggression. Therefore, a three-month follow-up interval was considered appropriate for examining the short-term reciprocal dynamics between personality perceptions and dating violence victimization.

An Instructional Manipulation Check (IMC) [48] was used to ensure data quality, resulting in the exclusion of 56 careless respondents. One individual who did not report their gender and another who had never been in a romantic relationship were also excluded. Furthermore, to control for potential confounding factors, 18 participants whose romantic partners were of the same gender, 7 who did not report their partner’s gender, 1 who self-identified as transgender, and 31 who did not respond to, or declined to answer questions about a previous marriage were excluded. As a result, the final analytic sample for T1 comprised 935 participants (328 men, 607 women; M_age = 24.31, SD = 3.22).

A follow-up survey (T2) was conducted three months later. Of the 935 eligible participants, 331 responded to the T2 survey (response rate: 35.4%). Among them, 290 respondents could be matched to their T1 survey data using participant IDs. To ensure that responses reflected the same romantic relationship across both waves, only those whose reported partner’s identifying letter matched at both T1 and T2 were retained, yielding 261 participants.

Data quality was reassessed at T2 using the IMC, and one additional careless respondent was excluded. After this step, 260 participants remained. Finally, participants whose romantic relationships had ended by T2 were excluded, yielding a final analytic sample of 206 individuals (62 men, 144 women; M_age = 24.87, SD = 3.13).

This sequential screening process ensured that the final sample consisted of individuals who (a) participated in both waves, (b) referred to the same romantic partner in both waves, (c) passed data quality checks at both T1 and T2, and (d) remained in their relationship throughout the study period.

### Data collection period

T1 took place from November 26 to December 12, 2021, and T2 occurred from February 25 to March 14, 2022.

### Questionnaire composition

Table 1 summarizes participants’ demographic and relationship characteristics, including relationship duration, frequency of contact, and relationship stage. A demographic questionnaire was used to collect participants’ age and gender. Basic questions about romantic relationships included relationship duration, partner’s gender, cohabitation status, frequency of contact, and relationship progress [49].

**Table 1:** Characteristics of relationships included in the analysis.

		n	%
Relationship Duration			
	1. (Within 1 month)	5	2.4
	2. (Within 2–3 months)	11	5.3
	3. (Within 6 months)	20	9.7
	4. (Within 9 months)	13	6.3
	5. (Within 1 year)	19	9.2
	6. (Within 1.5 years)	20	9.7
	7. (Within 2 years)	39	18.9
	8. (Approximately 3 years or more)	79	38.4
Frequency of Face-to-Face Conversations			
	0. (Once a month or less)	31	15.0
	1. (Approximately once every 2–3 weeks)	25	12.1
	2. (Approximately once a week)	39	18.9
	3. (Approximately 2–3 times a week)	25	12.1
	4. (Approximately 4–5 times a week)	15	7.3
	5. (Almost every day)	71	34.5
Stages of Romantic Progression			
	Stage 1 (Conversations, advice, exchanging gifts)	3	1.5
	Stage 2 (Dating, calling without specific reasons)	19	9.2
	Stage 3 (Introducing as boyfriend/girlfriend to friends; kissing; hugging)	18	8.7
	Stage 4 (Introducing as a romantic partner to friends and others)	70	34.0
	Stage 5 (Making marriage promises, proposing marriage)	96	46.6

Perceived Big Five personality traits were assessed using the 20-item Big Five Scale Short Version (BFS-S) developed by Uchida [50]. The BFS-S measures perceptions of others’ personality traits across five dimensions: agreeableness, neuroticism, conscientiousness, openness to experience, and extraversion. Participants used a 10-point scale (1 = not at all; 10 = very much) to respond to the following prompt: “How much does each of the following items apply to your current romantic partner?” Higher subscale scores indicated stronger perceptions of partners’ traits. Although the short version of this scale inevitably reduces item-level precision compared to full-length inventories, Uchida [50] demonstrated that the BFS-S can be applied as an other-rating measure and that its five-factor structure is preserved when used to assess impressions of others. Previous validation studies have also shown adequate internal consistency (αs = .72–.84). Based on this evidence, the BFS-S was adopted in this study to assess participants’ perceptions of their partners’ personality traits.

Perceptions of partners’ anger-related characteristics were assessed using the Japanese version of the MAQ [21,22], which comprises four subscales: aggression (external expression of anger), controlled affect (emotional regulation), guilt (internalized response to anger), and social inhibition (tendency to suppress anger in social contexts). Items were rated on a 5-point scale (1 = does not apply at all, 5 = applies very much), with higher scores indicating stronger perceptions of each trait in the respondents’ partners. The Japanese MAQ has demonstrated solid psychometric properties, with internal consistency ranging from α = .74 to.80 and test–retest reliability from r = .60 to.71 over a 16-week interval [22]. In the same study [22], its four-factor structure was confirmed through factor analysis, and criterion-related validity was supported by significant correlations with other established anger- and aggression-related measures. Furthermore, peer–self correlations were particularly high for the aggression subscale (r ≈ .76), indicating that these anger-related tendencies are readily observable and can be reliably evaluated by others. Based on this evidence, the Japanese MAQ was used in this study as an other-rating measure with which participants evaluated their partners’ typical anger expression styles to capture perception-based representations of anger-related traits.

Perceived attachment style was measured using the 30-item General Other version of the Intimate Relationship Experience Scale (ECR-GO) [28], based on Nakao and Kato’s [51] original scale. This scale was originally developed as a self-report measure in Nakao and Kato [51], but was later adapted into an other-rating format by Nakao [28] to enable assessment based on partner perceptions. The two subscales were “avoidance of intimacy” and “fear of abandonment.” The ECR-GO has demonstrated high internal consistency (α = .83–.90) and one-month test–retest reliability (r = .82–.92). Its two-factor structure—avoidance of intimacy and fear of abandonment—was confirmed through factor analysis, and criterion validity was established based on significant correlations with theoretically related constructs such as self-esteem. Furthermore, prior research [28] has shown that attachment-related tendencies can be recognized to some extent by others, supporting the use of the ECR-GO as an other-rating measure for assessing perceived attachment style in this study. Participants were instructed to rate each item on a 7-point scale (1 = not at all, 7 = very much), reflecting how they perceived their partners rather than predicting how their partners might self-report. Higher scores indicated greater perceived avoidance of intimacy or fear of abandonment in one’s partner.

Dating violence victimization was assessed using the six-item Indirect Violence Victimization from a Partner scale, created by Soma, Ura, Ochiai, and Fukazawa [52] and modified by Soma, Gushiken, and Ueda [53]. Participants were asked to rate their experiences on a 5-point scale (1 = never, 5 = very often). Higher total scores indicated more severe victimization by partners.

### Ethical considerations

Considering the sensitive nature of the survey, which addressed dating violence victimization, and the potential for emotional distress, several precautionary measures were implemented. First, the cover sheet included a notice informing participants that the questionnaire contained questions regarding violence. Second, informed consent was emphasized, and it was made clear that participants could withdraw at any time if they felt uncomfortable. Third, respondents could only proceed to the questionnaire if they agreed to participate by selecting “I agree,” which was considered to indicate written informed consent. Fourth, contact information for professional support services was provided on the cover and final pages of the questionnaire. The study was conducted with the approval of the Ethics Committee of the Graduate School of Education, Tohoku University (Approval No. 21-1-043).

### Analytic strategy

Analyses were conducted using SPSS (Ver. 29.0) and IBM SPSS Amos (Ver. 29.0). First, descriptive statistics and Pearson correlation coefficients were computed to examine the basic characteristics of the data and associations among variables.

Because the attrition rate from T1 to T2 was considerably high—partly due to the rigorous exclusion procedures (IMC failures, inconsistencies in partner identification, and relationship dissolution) and partly due to participants’ discontinuation of crowdsourcing platform use—it was necessary to verify that the final analytic sample did not exhibit systematic bias. In addition, dating violence victimization variables are known to show positively skewed distributions. Therefore, skewness was examined to assess the distributional properties of these variables and to ensure that model estimation would not be adversely affected by non-normality.

Next, longitudinal measurement invariance across T1 and T2 was examined for each scale. Configural invariance across waves was tested using confirmatory factor analysis (CFA), treating measurement occasions (T1 vs. T2) as groups, to assess whether factor structures were stable over time.

After these preliminary steps, structural equation modeling, including cross-lagged panel analysis, was conducted to investigate the reciprocal relationship between dating violence victimization and perceived partner characteristics. The model was estimated using the maximum likelihood method. Model fit was evaluated using standard indices: chi-square divided by degrees of freedom (χ²/df), goodness-of-fit index (GFI), adjusted goodness-of-fit index (AGFI), comparative fit index (CFI), root mean square error of approximation (RMSEA), and standardized root mean square residual (SRMR). Additionally, standardized path coefficients (β), model comparison indices (AIC), and residual covariances were reported.

To begin the model-building process, we tested a comprehensive model that included all 11 perceived personality traits measured in this study: the Big Five personality dimensions (extraversion, agreeableness, conscientiousness, neuroticism, and openness to experience), four anger-related coping traits conceptualized in Müller’s framework (aggression, controlled affect, guilt, and social inhibition), and two attachment style dimensions (fear of abandonment and avoidance of intimacy). Given the sample size and our aim to maintain theoretical clarity and statistical parsimony, we designated three focal traits for the final cross-lagged model based on the preceding theoretical and empirical literature. From the Big Five framework, we focused on agreeableness, which has been repeatedly associated with aggressive behavior and dating violence, particularly due to its interpersonal implications such as trust, empathy, and cooperation [16–20]. Among anger-related traits, we selected aggression—the outward expression of anger—as it has consistently emerged in both Western and Japanese research as a strong predictor of dating violence [23–25]. Finally, from attachment theory, we included fear of abandonment, which reflects anxiety-driven control behaviors in close relationships and has shown predictive utility for dating violence in longitudinal studies [34,35]. By narrowing the model to these three representative traits—agreeableness, aggression, and fear of abandonment—we aimed to balance theoretical comprehensiveness with statistical parsimony. This focused approach allowed us to test the core hypotheses regarding reciprocal relationships between perceived partner traits and dating violence victimization while avoiding potential problems of multicollinearity and model overcomplexity in the full-variable specification. Model simplification was considered when appropriate, using standard fit indices and theoretical interpretability.

In the model-building process, bidirectional cross-lagged paths were specified from T1 perceived traits to T2 dating violence victimization and from T1 dating violence to T2 perceived traits. In the initial model, covariances were freely estimated among all T1 variables (i.e., dating violence victimization and perceived personality traits at T1) to account for their concurrent associations at baseline. Gender was included as a control variable, as were relationship duration and frequency of face-to-face communication, based on the premise that perceptions of others’ personality characteristics are shaped by repeated interpersonal interactions [44]. Relationship duration and frequency of face-to-face communication were assessed at both time points. Considering the strong correlations between T1 and T2 scores and to avoid potential multicollinearity, only T1 values were entered as baseline controls in the model, consistent with the analytical approach of Kanemasa et al. [35]. During model refinement, non-significant paths were removed to improve parsimony, and selected residual covariances between the T2 error terms were introduced to account for shared variance not fully captured by the structural paths. In a subsequent refinement step, paths from the control variables were specified where appropriate to reflect baseline associations and improve model fit.

Finally, multicollinearity among variables included in the model was evaluated using variance inflation factors (VIFs), condition indices, and variance–decomposition proportions to ensure that parameter estimates were stable and interpretable.

## Results

### Descriptive statistics and preliminary analyses

Means, standard deviations, and reliability coefficients (Cronbach’s α) for each scale used in this study, including the perceived Big Five personality traits, four anger-related traits (aggression, controlled affect, guilt, and social inhibition), attachment style (avoidance of intimacy and fear of abandonment), and dating violence victimization, were calculated at both T1 and T2 (Table 2). All scales demonstrated adequate internal consistency, with Cronbach’s α values exceeding.70. Pearson correlation coefficients were calculated to clarify the interrelations among the variables (Table 3).

**Table 2:** Descriptive statistics of each variable.

	T1			T2		
	M	SD	α	M	SD	α
Dating Violence Victimization	11.50	5.23	.865	11.44	5.34	.884
Extraversion	27.71	7.37	.829	27.30	7.06	.806
Agreeableness	22.11	8.54	.848	21.84	8.48	.847
Neuroticism	31.63	6.18	.848	31.25	6.00	.839
Openness to Experience	26.05	7.33	.780	25.77	7.66	.747
Conscientiousness	25.56	6.53	.802	25.54	6.24	.837
Aggression	13.91	6.45	.890	14.02	6.42	.887
Social Inhibition	17.51	5.22	.804	17.42	5.05	.798
Guilt	16.99	5.17	.801	17.03	5.53	.839
Controlled Affect	12.11	3.57	.764	11.89	3.64	.797
Avoidance of Intimacy	46.81	12.09	.838	46.84	10.56	.780
Fear of Abandonment	52.91	18.74	.896	52.09	19.49	.905

**Table 3:** Pearson correlations among selected variables in the final model.

	1	2	3	4	5	6	7	8	9	10	11
1. Gender	―	−.03	.05	−.11	.17*	.00	−.26***	−.18**	.16*	.02	−.36***
2. Frequency of Face-to-Face Conversations		―	.11	.16*	−.01	.20**	.05	.15*	.04	.22**	−.02
3. Relationship Duration			―	.11	.00	.10	−.08	.04	−.05	.13	−.04
4. T1 Dating Violence Victimization				―	−.57***	.68***	.42***	.82***	−.49***	.67***	.43***
5. T1 Perceived Agreeableness					―	−.53***	−.25***	−.54***	.74***	−.49***	−.32***
6. T1 Perceived Aggression						―	.34***	.64***	−.46***	.80***	.39***
7. T1 Perceived Fear of Abandonment							―	.40***	−.25***	.34***	.69***
8. T2 Dating Violence Victimization								―	−.53***	.71***	.45***
9. T2 Perceived Agreeableness									―	−.48***	−.30***
10. T2 Perceived Aggression										―	.39***
11. T2 Perceived Fear of Abandonment											―

*p <.05, **p <.01, ***p <.001. Gender was coded as 0 = men and 1 = women.

Potential attrition bias was assessed using independent-samples t-tests and estimated effect sizes (Hedges’ g) to calculate the differences between participants who completed both waves (n = 206) and those who dropped out after T1 (n = 729). The differences were minimal for most variables, with |g| < .20 for neuroticism, agreeableness, conscientiousness, openness, anger-related traits, attachment dimensions, and dating violence victimization. The results showed small effects for extraversion (g = .17, slightly higher among retained participants) and social inhibition (g = −.21, slightly higher among dropouts), and a marginal difference for avoidance of intimacy (g = −.16). Overall, differences were generally small; however, modest selective attrition cannot be ruled out. Importantly, for the variables included in the final model (agreeableness, aggression, fear of abandonment, and dating violence victimization), differences between retained participants and dropouts were small (all |g| < .20), suggesting that attrition-related bias in the primary analyses is likely limited.

The skewness values for T1 and T2 victimization were 0.81 and 0.93, respectively, indicating a mild positive skew. However, these values were within the acceptable range. Considering the sample size (n = 206) and robustness of maximum likelihood estimation to moderate non-normality, data transformation was not applied. Thus, the model estimation was considered stable.

### Measurement invariance across time

To examine the stability of measurement structures across waves, a series of multigroup CFAs were conducted for each scale.

For the Indirect Violence Victimization from a Partner scale, the six-item single-factor model showed good fit at both T1 (χ²/df = 2.09, CFI = 0.986, RMSEA = 0.073) and T2 (χ²/df = 1.89, CFI = 0.990, RMSEA = 0.066). When tested simultaneously, the configural model also showed satisfactory fit (χ²/df = 1.99, CFI = 0.988, RMSEA = 0.049, SRMR = 0.029), indicating that the factor structure was stable across waves. In this model, a residual covariance was specified between Item 4 and Item 6 at both waves to account for their similar content (behavioral restriction/management). These results indicate that the basic factor structure was stable across waves, providing some support for the comparability of the scale across time.

For the Japanese MAQ, model fit was acceptable—though not ideal—at both time points (T1: χ²/df = 2.94, CFI = 0.81, RMSEA = 0.097, SRMR = 0.112; T2: χ²/df = 2.59, CFI = 0.84, RMSEA = 0.088, SRMR = 0.103). The configural model across waves also fell within an acceptable range (χ²/df = 2.76, CFI = 0.83, RMSEA = 0.066, SRMR = 0.111). These results suggest that the four-factor structure remained stable over time, supporting configural invariance despite some suboptimal indices (e.g., SRMR).

For the Big Five Scale, model fit was acceptable at each wave (T1: χ²/df = 2.16, CFI = 0.89, RMSEA = 0.075, SRMR = 0.075; T2: χ²/df = 2.79, CFI = 0.85, RMSEA = 0.093, SRMR = 0.091). The configural model including both time points also showed acceptable fit (χ²/df = 2.47, CFI = 0.87, RMSEA = 0.060, SRMR = 0.075). These findings support configural invariance and suggest that longitudinal changes in perceived partner traits represent genuine perceptual shifts rather than measurement artifacts.

For the ECR-GO, model fit was poorer at each time point (T1: χ²/df = 3.51, CFI = 0.65, RMSEA = 0.111; T2: χ²/df = 3.74, CFI = 0.64, RMSEA = 0.116). However, the overall factorial pattern was largely consistent over time, suggesting that the observed poor fit may reflect limitations of the measurement model in the present sample (e.g., item-level misfit and/or method effects). Accordingly, results involving fear of abandonment should be interpreted cautiously. Moreover, the internal consistency coefficients for the fear of abandonment and avoidance of intimacy dimensions were sufficiently high (αs > .83). Considering the theoretical relevance of attachment style to this study and that the structure remained conceptually stable across measurement waves, the scale was retained for subsequent analyses with appropriate caution. Because baseline CFA fit was poor, we could not test stronger forms of longitudinal invariance; therefore, cross-lagged findings involving fear of abandonment should be interpreted cautiously.

### Structural equation modeling

We first evaluated an exploratory comprehensive model including all 11 traits. This model showed poor fit (χ²/df = 3.042, GFI = 0.778, AGFI = 0.691, CFI = 0.813, RMSEA = 0.100, SRMR = 0.121). This inadequate fit could be due to excessive model complexity, which increases the number of estimated parameters relative to the sample size, and overlap among conceptually related constructs, especially within the anger-related subscales and Big Five traits. Together, these features may limit the ability to detect unique effects and may be associated with less precise estimates (e.g., larger standard errors).

Accordingly, consistent with our a priori analytic plan, we proceeded with the primary model including agreeableness, aggression, and fear of abandonment. By narrowing the model scope to these three representative traits—agreeableness, aggression, and fear of abandonment—we aimed to balance theoretical comprehensiveness and statistical parsimony. This shift allowed us to move from an overparameterized model to a theoretically coherent and statistically stable specification. This focused approach allowed us to test the core hypotheses regarding reciprocal relationships between perceived partner traits and dating violence victimization while avoiding the problems of multicollinearity and model overcomplexity observed in the full-variable model.

In Model 1, the model fit was not satisfactory (χ²/df = 3.546, GFI = 0.898, AGFI = 0.827, CFI = 0.911, RMSEA = 0.111, SRMR = 0.093). In Model 2, non-significant paths were removed to simplify the model and reduce complexity. This pruning slightly improved model parsimony (AIC = 167.04 vs. 192.28); however, the overall model fit remained suboptimal (χ²/df = 2.876, GFI = 0.912, AGFI = 0.854, CFI = 0.933, RMSEA = 0.096, SRMR = 0.095), suggesting that the model lacked key explanatory connections.

Subsequent model refinement was guided by both statistical criteria and theoretical considerations. Non-significant paths were removed in the early stage (Model 1 → Model 2), whereas later modifications (Model 2 → Model 3) were informed by established findings on interpersonal processes and partner perception. Model 3 incorporated additional paths and covariances to achieve further improvement. Specifically, covariances were added between the error terms for T2 dating violence and T2 perceived aggression, as well as between T2 dating violence and T2 perceived agreeableness. These covariances were included based on the assumption that the variables share similar underlying psychological dimensions, such as emotional reactivity or interpersonal conflict, that may not be fully accounted for by direct causal paths. Furthermore, direct paths were added from gender to T1 perceived agreeableness and T1 perceived fear of abandonment, as well as from conversation frequency to T1 dating violence and T1 perceived aggression based on their significant associations in preliminary regression analysis. Covariance was also introduced between T1 perceived aggression and T1 dating violence to reflect shared variance between these concurrent variables.

These modifications substantially improved model fit (χ²/df = 2.269, GFI = 0.936, AGFI = 0.884, CFI = 0.959, RMSEA = 0.079, SRMR = 0.065), with a lower AIC (141.67), indicating that Model 3 provided the best representation of the data. Table 4 summarizes the model construction process and fit indices for each model. Model 3 demonstrated superior fit compared to Models 1 and 2, supporting the inclusion of both residual covariances and theoretically supported control paths.

**Table 4:** Construction process of the cross-lagged panel model in this study.

Model	χ²/df	GFI	AGFI	CFI	RMSEA	SRMR	AIC
Model 1 (Initial)	3.546	0.898	0.827	0.911	0.111	0.093	192.28
Model 2	2.876	0.912	0.854	0.933	0.096	0.095	167.04
Model 3 (Final)	2.269	0.936	0.884	0.959	0.079	0.065	141.67

The major standardized path coefficients in the final model were as follows. The path from T1 dating violence to T2 dating violence was β = 0.712 (p < .001). The path from T1 perceived aggression to T2 perceived aggression was β = 0.637 (p < .001), and that from T1 perceived fear of abandonment to T2 perceived fear of abandonment was β = 0.621 (p < .001). The path from T1 perceived agreeableness to T2 perceived agreeableness was β = 0.729 (p < .001), demonstrating the strong temporal stability of perceived partner traits.

Regarding cross-lagged effects, T1 dating violence significantly predicted T2 perceived aggression (β = 0.241, p < .001). T1 dating violence also predicted T2 perceived fear of abandonment (β = 0.168, p < .01). Conversely, T1 perceived aggression predicted T2 dating violence (β = 0.152, p < .01), highlighting a bidirectional relationship between dating violence victimization and perceived partner aggression.

Among the control variables, gender significantly predicted T1 perceived agreeableness (β = 0.144, p < .01) and T1 perceived fear of abandonment (β = –0.241, p < .001), while conversation frequency predicted T1 dating violence (β = 0.132, p < .05) and T1 perceived aggression (β = 0.180, p < .001).

**Fig 1:**
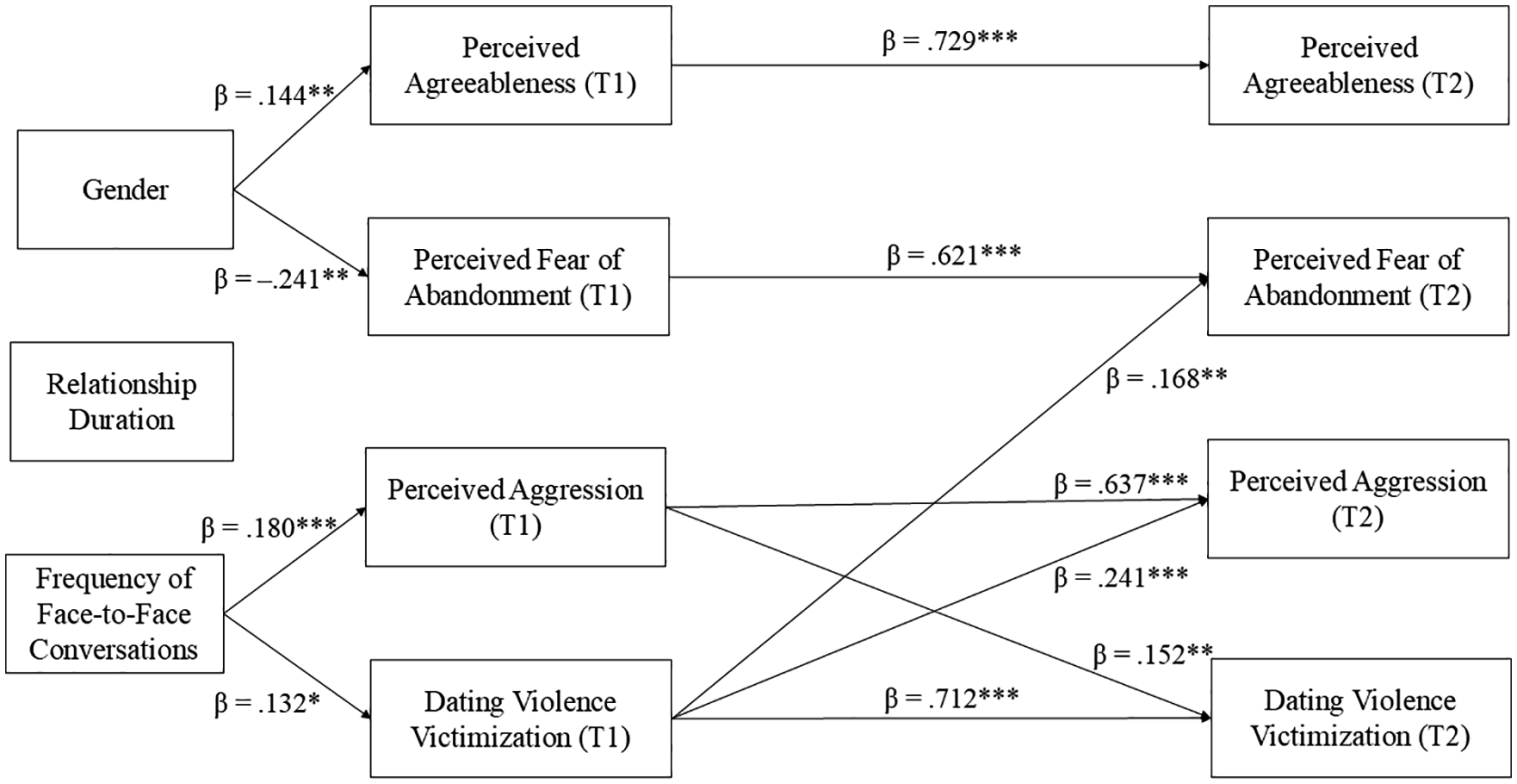
Process of reciprocal influence between dating violence victimization and personality perceptions of dating partners. All coefficients are standardized. Solid arrows indicate statistically significant paths (*p < .05, **p < .01, and ***p < .001). For clarity, some residual covariances are omitted from the diagram but were included in the model. Specifically, residual covariances were estimated between T2 dating violence victimization and T2 perceived aggression (r = .324, p < .001), as well as between T2 dating violence victimization and T2 perceived agreeableness (r = –.157, p < .05). Covariances were also estimated among all T1 variables to reflect their intercorrelations. For example, at T1, perceived agreeableness was negatively associated with perceived fear of abandonment (r = –.221, p < .01), perceived aggression (r = –.556, p < .001), and dating violence victimization (r = –.569, p < .001). Similarly, perceived fear of abandonment at T1 was positively correlated with perceived aggression (r = .365, p < .001) and dating violence victimization (r = .404, p < .001), and perceived aggression at T1 was positively associated with dating violence victimization (r = .669, p < .001).

Overall, these results indicated bidirectional cross-lagged associations between dating violence victimization and perceptions of partners’ personality characteristics. Specifically, higher dating violence victimization at T1 predicted higher perceived aggression and fear of abandonment in partners at T2. Conversely, higher perceived aggression at T1 predicted greater dating violence at T2. The final model showed good fit, confirming its suitability for the data (Fig 1).

### Multicollinearity diagnostics

To assess potential multicollinearity, VIFs were examined for all predictors. VIF values ranged from 1.05 to 2.29, well below the conventional threshold of 5.0 (and the stricter criterion of 2.5). The condition indices were below 30, and no evidence of variance inflation across multiple predictors was observed. Therefore, multicollinearity was not considered a problem in the present model, and the parameter estimates could be interpreted as stable.

## Discussion

This study examined the relationship between dating violence victimization and perceptions of dating partners’ personalities, focusing on both directional and reciprocal influences. A longitudinal investigation was conducted on three personality characteristics—the Big Five traits, aggression, and attachment style—to determine how victims’ perceptions of these characteristics are related to dating violence. The results indicated different patterns of temporal and reciprocal associations for each type of perceived personality characteristic.

Regarding the relationship between dating violence victimization and perceived Big Five traits, the covariance at T1 and error covariance at T2 in the cross-lagged model revealed a significant negative association between dating violence victimization and agreeableness. A previous theory posited a negative relationship between agreeableness and aggression [15], and the current findings align with this view. Low agreeableness is considered one of the most significant Big Five predictors of aggression [16], and this link was also apparent in this study. Thus, distrust of others could lead to aggression in conflict situations [17], and victims may perceive such tendencies. Although the cross-sectional relationships between the Big Five traits and dating violence were robust, no longitudinal influence was observed, implying that the former may be more associated with concurrent or coexisting aspects of dating violence victimization. One reason for this could be that the Big Five is a basic personality theory that focuses on relatively stable characteristics [11]. Another possibility involves interpersonal interactions. Personality perceptions are shaped by social exchanges as relationships grow [44]. Considering that most participants had already been in relationships for over a year (with many at a relatively advanced stage) [49], their perceptions of fundamental traits may have stabilized, thereby reducing the likelihood of observing longitudinal effects. In addition, the absence of longitudinal effects for the Big Five traits may reflect methodological limitations. The short 20-item scale used in this study, while efficient and widely validated, may be less sensitive to subtle intra-individual changes over relatively brief intervals than longer measures. Future studies using full-length or facet-level measures may more effectively capture dynamic shifts in perceived partner personality following experiences of dating violence.

Regarding the relationship between dating violence victimization and perceived partner aggression, the paths from T1 victimization to T2 perceptions of aggression and from T1 perceptions of aggression to T2 victimization were significant, indicating a reciprocal influence. The idea that personality perceptions may predict future victimization and that victimization may shape personality perceptions has been supported. Victims appear to have some awareness of their partners’ tendency to outwardly express anger, which is reinforced when actual violence occurs. Considering that aggression is frequently cited as a major predictor of dating violence [23 –26], these findings suggest that even victims’ perceptions of aggression can predict future violence. Aggression may be more easily observed than other traits, allowing victims to recognize it to some extent before experiencing violence.

However, the effect sizes of the cross-lagged paths were modest in magnitude (β = 0.15–0.24). Therefore, these associations should be interpreted as incremental rather than dominant influences. From a practical standpoint, this suggests that although perceptions of aggression may contribute to subsequent victimization, they likely operate alongside many other interpersonal or contextual factors that influence relationship dynamics. Thus, the results highlight a subtle but meaningful process in which victims’ perceptions and experiences of aggression reinforce each other over time rather than indicating a strong causal loop.

Regarding the relationship between dating violence and perceived attachment style, analyses focusing on fear of abandonment revealed that T1 dating violence predicted T2 perceptions of fear of abandonment but not the reverse. One possible explanation for this is that victims may find it difficult to assess perpetrators’ fear of abandonment. Although perpetrators’ self-reported fear of abandonment has been linked to future violence [35], it did not emerge as a predictor when based on victims’ perceptions in this study. Nakao [28] found that self-reported attachment style and attachment style as perceived by others are correlated, albeit not strongly, suggesting that attachment style may not be as externally evident as aggression. Another explanation is that victims’ understanding of perpetrators’ attachment styles involves interpretive processes. Fear of abandonment has been theorized to drive attempts to control one’s partner [31]. When victimization prospectively predicted higher perceived fear of abandonment in perpetrators, it implies that victims interpret the violence as stemming from underlying anxiety. In this sense, victims’ perceptions of perpetrators’ attachment style may arise as a meaning-making process related to violence.

However, this prospective association should be interpreted with caution. In the present data, the ECR-GO showed suboptimal CFA fit at both waves (CFI < .70), which suggests that the intended two-factor structure may not adequately reproduce the observed covariance structure in this sample. Although the factorial pattern appeared broadly similar across time and internal consistency was high, limited model fit raises the possibility that changes in observed scores partly reflect measurement artifacts (e.g., item-level misfit or method effects) rather than purely substantive shifts in perceived attachment-related tendencies. Accordingly, the finding that victimization predicted later perceptions of fear of abandonment should be interpreted as tentative and in need of replication with improved measurement, and future studies should re-examine this process using improved measurement (e.g., revised or shorter other-rating items, self–partner multi-informant designs, and/or alternative modeling approaches that better capture the scale structure).

### Implications for clinical practice

The findings of this study offer several insights for supporting victims of dating violence. First, they may inform preventive interventions. Previous longitudinal research has shown that certain perpetrator personality traits predict future violence [35], suggesting that examining these traits from the perspective of victims may help prevent dating violence [43]. This study found that among the traits perceived by victims, only aggression predicted victimization three months later. Therefore, focusing on aggression, which is comparatively overt, may be more effective than focusing on the Big Five traits or attachment style, which may be more interpretive. Identifying which traits perceived by victims predict future violence could enhance preventive interventions.

Second, this study clarifies the nature of victims’ perceptions. The findings revealed that experiencing dating violence victimization was associated with victims perceiving perpetrators as both “aggressive” and “afraid of being abandoned.” This shift indicates that victims do not merely view perpetrators as violent but also as individuals driven by anxiety over possible abandonment. Understanding how victims conceptualize their perpetrators’ personalities could be useful in counseling or support settings and may contribute to a more nuanced approach to victim care.

### Study significance and future directions

Using a longitudinal approach, this study investigated the relationship between dating violence victimization and victims’ perceptions of perpetrators’ personalities. Three key conclusions emerged. First, among the Big Five traits, low agreeableness correlated with victimization, but only cross-sectionally. Second, perceived aggression and dating violence victimization showed reciprocal influence, with victims’ perceptions of aggression predicting victimization three months later. Third, victimization predicted subsequent perceptions of fear of abandonment in perpetrators.

However, this study has some limitations. Most participants had been in long-term relationships for three or more years. Considering that personality perceptions evolve through interactions [44], further research focusing on couples in the early stages of relationships, when perceptions may change more substantially, is required. Another limitation concerns the type of violence measured. The scale used in this study [53] primarily assessed psychological violence; however, dating violence can be physical, economic, or sexual [2]. Different forms of violence may vary in how they influence victims’ perceptions of perpetrators’ personalities; therefore, a broader approach is necessary in the future. The third issue is the link between victims’ perceptions and coping behaviors. Fear of abandonment may lead to situations in which victims have trouble leaving perpetrators or resort to engaging in codependent communication; thus, exploring whether victims’ personality inferences affect their coping strategies or intentions to continue a relationship will be worthwhile.

Another methodological limitation concerns the duration and platform of the longitudinal study. Although a three-month interval was adopted for the longitudinal design, it remains unclear whether this period was optimal for capturing meaningful changes in personality perceptions and experiences of dating violence. Future studies may benefit from exploring both shorter and longer timeframes to assess the temporal dynamics of these variables more effectively.

Furthermore, all variables in this study were based on victims’ perceptions of their partners’ traits and behaviors. Thus, the findings may reflect subjective interpretations rather than perpetrators’ objective characteristics. Previous validation research on the Japanese MAQ [22] demonstrated that peer–self correlations were considerably higher for the aggression subscale (r ≈ .76) than for other subscales (controlled affect, guilt, and social inhibition), indicating that aggression is a more observable dimension. Therefore, the present finding that perceived aggression was the most stable and robust predictor may partly reflect this difference in observability. Thus, externally visible traits are more readily captured through perception-based assessments, whereas more internalized aspects such as emotional regulation or guilt may have been underestimated because of the inherent difficulty of external evaluation. Future research combining self- and partner-reports would help clarify whether these differences represent true psychological independence or perceptual bias.

In addition, although the present findings identified reciprocal associations between perceived aggression and dating violence victimization, these effects should not necessarily be interpreted as direct causal loops. Alternative explanations such as shared contextual or situational factors, including stress, conflict frequency, or communication problems, may simultaneously increase perceived aggression and victimization experiences. Incorporating third variables such as this in future longitudinal designs would help clarify whether the observed reciprocity reflects true bidirectional causality or common underlying relationship stress processes. Moreover, the lack of longitudinal effects for the Big Five traits may reflect the relative temporal stability of broad personality dimensions and limited sensitivity of short-form measures to detect subtle within-person changes over short intervals.

Finally, this study had a relatively high attrition rate between T1 and T2, and participants were recruited from a single crowdsourcing platform (CrowdWorks). Although completer–dropout comparisons suggested only small differences on observed variables, selective retention cannot be ruled out. In particular, the combination of (a) intermittent participation patterns typical of crowdsourcing platforms and (b) strict inclusion criteria to ensure data quality and relationship continuity may have overrepresented respondents with more stable online engagement and higher survey compliance. Accordingly, the present findings may generalize most directly to young adults who are willing and able to participate repeatedly online and who remained in the same relationship over the follow-up period. Future studies using multiple recruitment sources and strengthened retention strategies (e.g., mixed-mode follow-up and enhanced incentives/tracking) are needed to evaluate the robustness and broader representativeness of these longitudinal associations.

## Supporting information

S1 Data Dataset for the longitudinal analyses examining the reciprocal influence between dating violence victimization and perceptions of partners’ personality traits. (XLSX)
